# Prevalence and molecular identification of zoonotic *Anisakis* and *Pseudoterranova* species in fish destined to human consumption in Chile

**DOI:** 10.1007/s00436-022-07459-x

**Published:** 2022-03-01

**Authors:** Tamara Muñoz-Caro, Alvaro Machuca, Pamela Morales, Javiera Verdugo, Rodrigo Reyes, Macarena García, Liliana Rutaihwa, Tobias Schindler, Sven Poppert, Anja Taubert, Carlos Hermosilla

**Affiliations:** 1 Escuela de Medicina Veterinaria, Facultad de Medicina Veterinaria Y Recursos Naturales, Universidad Santo Tomás, Talca, Chile; 2 Swiss Tropical and Public Health Institute, Basel, Switzerland; 3 Institute of Parasitology, Justus Liebig University Giessen, Giessen, Germany

**Keywords:** *Anisakis*, *Pseudoterranova*, Fish-borne zoonoses, Anisakiasis

## Abstract

Zoonotic larvae of the family Anisakidae found in several fish species represent a serious risk in public health since they may cause food-borne anisakidosis in humans. Chile has culinary preferences including eating raw fish in many traditional preparations. In the present study, a total of 180 fish specimens representing three different fish species, i.e., Chilean hake (*Merluccius gayi*), snoek (*Thyrsites atun*), and sea bream (*Brama australis*), were caught at central coast of Chile. Parasitological examination was performed on musculature and abdominal cavity for subsequent extraction and quantification of anisakid larvae. Estimation of infection parameters, such as prevalence, was performed indicating 100% (CI: 0.94–1.0) prevalence of anisakid L3 in Chilean hakes and snoeks*.* Moreover, sea breams reached a prevalence of 35% (CI: 0.23–0.48). Prevalence of anisakid larvae in muscle was also analyzed showing values of 18.6% (CI: 0.097–0.309) in Chilean hakes, 15% (CI: 0.07–0.26) in snoeks, and 1.7% (CI: 0–0.089) in sea breams. Meanwhile, prevalence of anisakid larvae in internal organs showed highest values for peritoneum (100% and 83.3%) for snoeks and Chilean hakes, respectively, for liver (96.7%) and gonads (86.6%) in Chilean hakes, and for intestine (98.3%) in snoeks. Molecular analysis of collected anisakid L3 unveiled presence of two potentially zoonotic nematode species, i.e., *Pseudoterranova cattani* and *Anisakis pegreffii*. *P. cattani* was found in Chilean hakes and snoeks being the first molecular host species report for Chilean snoeks. Besides, *A. pegreffii* was also identified in these species being the first molecular report on this regard. These findings are relevant for better understanding of epidemiology of anisakiasis in Chilean coasts and for public health issues considering potential risk of human population due to its culinary preferences in eating raw fish.

## Introduction

Helminth nematodes of the family Anisakidae have a worldwide distribution and present a complex heteroxenous lifecycle in marine habitats including various intermediate host species (Koie et al. 1995). Several anisakid nematodes have been found as adult stages parasitizing the digestive tract of marine mammals including seals, sea lions, sea leopards, sea elephants, baleen, and toothed cetaceans (Hermosilla et al. 2016; Mattiucci et al. 2018; Ebmer et al. 2020). Conversely to adult stages, anisakid larval stages are often found parasitizing the body cavity and muscle of many obligate intermediate/paratenic host fish species and cephalopods (Chai et al. 2005; Mattiucci et al. 2018). Thus, anisakid third-stage larvae (L3) represent a potential risk to humans as they may cause a neglected fish-borne zoonotic disease, known as gastric/intestinal anisakidosis, with main etiologic species belonging to the genera *Anisakis* and *Pseudoterranova* (Audicana et al. 2002; Mattiucci et al. 2013a,b). Human anisakiasis occurs when raw, undercooked, or marinated fish, containing living L3, are consumed, and thus represent a risk for human health worldwide (McClelland 2002; Zhu et al. 2002; Mattiucci and Nascetti 2008). Most cases of human anisakidosis have been reported to occur in Japan, Spain, the Netherlands, and Germany (Pravettoni et al. 2012). In South America, human anisakidosis is still considered as neglected re-emerging disease of public health concern (Eiras et al. 2018), with cases reported from Chile and Peru, both countries allocated alongside Southern Pacific coast and with abundant fish consumption (Barriga et al. 1999; Torres et al. 2000a, b; Mercado et al. 2006; Jofré et al. 2008, Aco Alburqueque et al. 2020). In most cases, the etiological agent was not identified at the species level (Aco Alburqueque et al. 2020).

Among the nine nominal species of *Anisakis*, only *A. simplex* (s.s.) and *A. pegreffii* are hitherto reported as causative agents of human anisakiasis (reviewed in Mattiucci et al. 2017). *Anisakis pegreffii* may cause gastric, intestinal, and gastro-allergic anisakiasis (GAA). Indeed, the parasite has been identified in invasive cases of anisakiasis, after observing viable larvae during endoscopy and/or colonoscopy (Mattiucci et al. 2013b; Lim et al. 2015), or in surgically removed eosinophilic granulomas (Mattiucci et al. 2011; Mladineo et al. 2016). Similarly, *A. simplex* (s.s.) has mostly been associated with invasive cases of gastric and intestinal anisakiasis rather than cases of GAA (Arai et al. 2014; Lim et al. 2015). Regarding *Pseudoterranova* infections in humans, clinical symptoms caused by these species seem to be weaker than those observed in *A. simplex* (s.s.) or *A. pegreffii* infections (Mattiucci et al. 2013b). In addition, this genus has not been reported to induce eosinophilic granulomas, even though weak infections of non-penetrating larvae may be accompanied by vomiting and gastric or intestinal pain (Cavallero et al. 2016). Furthermore, allergic symptoms associated with *Pseudoterranova* infections may have arisen from cross-reactions to secondary infections, after previous exposure to *A. simplex* (s.l.) larvae (Mattiucci et al. 2017). Among the *P. decipiens* complex, *P. cattani* has been reported to occur in Chile in three coastal fish species acting as paratenic/intermediate hosts, i.e., Chilean hakes, black cusk-eel (*Genypterus maculatus*), and corvina drum (*Cilus gilberti*) (George-Nascimento and Urrutia 2000), all of them found in popular Chilean fish dishes. Human cases due to *P. cattani* infections have also been reported in Chile (Weitzel et al. 1996), causing unusual oropharygeal pseudoterranovosis. However, most infections will be mild and thus non-reported. Moreover, few cases of human anisakiasis have been reported in Chile with no evidence of the *Anisakis* species causing pathology (Torres et al. 2000a, b). *A. simplex* larvae have been found in Chilean hakes (Torres et al. 2000a, b) and other fish species, such as Chilean jack mackerel (*Trachurus murphyi*) and small-eye flounder (*Paralichthys microps*) (Mercado et al. 2001; Torres et al. 2014). Interestingly, until date, no records exist on *A. pegreffii* infections in central coast fish species from Chile.

From a public health perspective, it is urgent to determine not only the exact prevalence of zoonotic anisakid larvae in paratenic hosts including fish and cephalopods destined to human consumption, but also parasitic burdens in order to estimate potential risks to consumers and propose adequate prophylactic measurements.

In this context, in Chile, the food sanitary code number 323 establishes that all fish for human consumption must be free of parasites and without cysts, and that all fresh and chilled fish that are sold or processed must be eviscerated as soon as they are caught, except for some species of reduced size (sardines, silversides, anchovies, and others). However, sanitary inspection of fish species extracted by artisanal fishery destined to human consumption in Chile varies with respect to industrial fishery of Atlantic salmon (*Salmo salar*) where traceability of final product in aquaculture farms exists. Therefore, it is hard to ensure in artisanal fishery whether all sanitary processes and good hygiene practices from capture to the fish sale are undertaken.

In the present epidemiological study, three main consumable fish species in Chile have been selected for parasitological and molecular analyses of possible anisakid L3 infections: the Chilean hake (*Merluccius gayi*; Guichenot 1848), the snoek (*Thyrsites atun*; Euphrasen 1791), and the sea bream (*Brama australis*; Valenciennes 1838)*.* As already stated, these fish species are very popular within Chilean gastronomy and frequently consumed by humans. More importantly, snoeks (*T. atun*) and sea breams (*B. australis*) are frequently eaten as raw meat in traditional culinary preparations such as “ceviche,” “sushi,” and “carpaccio.”

Infection parameters, such as prevalence, mean intensity (mI; parasitic burden), and mean abundance (mA) (according to Bush et al. 1997) as well as fish-internal anisakid larval distribution were analyzed in selected fish species. In addition, molecular analysis of collected anisakid L3 was conducted. Accordingly, generated data intents to provide scientific information on epidemiological and public health issues related to these neglected food-borne zoonotic diseases.

## Material and methods

### Study area and sample collection

During August 2019, a total of 180 fish belonging to three different local species, namely Chilean hake (*M. gayi*), snoek (*T. atun*), and sea bream (*B. australis*), were captured in two main artisanal fishing spots of Maule coast, i.e., Constitución and Iloca (coordinates 35°20′00″S 72°25′00″O and 34°55′00″S 72°11′00″O, respectively). All specimens (*n* = 180) were directly purchased from local markets of the same fishing locations. Fish were immediately transported at cooling conditions (4 °C) to laboratory facilities of Veterinary School of Saint Thomas University (UST) of Talca, Chile. Obtention of fresh specimens was carried out three times by using a convenient sample size of *n* = 60 per fish species and sampling procedure was conducted in less than 24 h after fish capture. The sample size of 60 specimens per fish species was chosen as the best compromise between a suitable estimation of parasitological indicators of prevalence, mI, and mA as well as sample costs according to previous reports (Jovani and Tella 2006, Marques and Cabral 2007, Bernardi et al. 2011 and Shvydka et al. 2018).

### Parasitological examination

In the laboratory facilities of UST in Talca, Chile, initial morphometrical data for each fish species were recorded including total length (*L*) and total body weight (BW). Subsequently, in all fish examined, skin was removed and musculature separated into epi-axial and hypo-axial by longitudinal dissection in thin cuts of maximum 4 mm thickness. Parasite load of each dissection was analyzed by using the trans-illumination technique allowing an adequate detection and isolation of anisakid larvae (Power 1958; Torres and Puga 2011) being likewise applied in Chile to export fishery products.

Thereafter, for each fish, the body cavity was opened carefully ventrally along the middle line for visual inspection and collection of free larvae present at the peritoneum (specifically, larval stages found on surfaces of peritoneal membranes within abdominal cavity). Subsequently, the selected internal organs, i.e., liver, stomach, gonads, and intestine, were carefully collected and placed into Petri dishes containing saline solution to perform further inspection and extraction of anisakid larvae under stereomicroscopy (Motic) analysis by using non-traumatic forceps. Total larvae collected either free in peritoneum or from internal organs were rinsed with distilled water and immediately transferred into 2-mL plastic tubes (Eppendorf), preserved in 70% ethanol until further molecular DNA analysis. Larvae were examined using light microscopy analysis (Motic) and a digital camera (Motic) for pre-identification of *Anisakis* and *Pseudoterranova* larvae based on morphological features (Hernández-Orts et al. 2013; Chen and Shih 2015). Fish collection and all sampling procedures were approved by the Animal Ethic Committee of the UST and conducted in accordance to current Chilean Animal Laws.

### Molecular analysis of anisakid larvae

Molecular analysis was performed by the Swiss Tropical Institute in Basel, Switzerland, and the Institute of Parasitology of the Justus Liebig University Giessen in Germany. For molecular analyses, a total of 5 larvae were collected from each location/organ of the three fish species: i.e., muscle, liver, stomach, intestine, and peritoneum. Thereafter, by taking equal number of larvae in all organs analyzed, a subsample of 13 anisakid larvae were used to generate sequences including a positive control (DNA from *Pseudoterranova cattani*).

Then, to identify fish-derived anisakid larvae, we generated and compared sequence data of mitochondrial cytochrome oxidase subunit 1 gene (mtDNA*cox1*) with GenBank database entries. Genomic DNA was isolated using the QiaAmp DNA Mini Kit® according to manufacturer’s protocol for tissue samples (Qiagen, Hilden, Germany). A short fragment of the mtDNA *cox1* gene was amplified using the following primer combinations: JB3 and JB4.5 (Bowles et al. 1992). We performed PCR with 50 µL reaction solution thereby using HotStar Taq Plus Master Mix®, 25 mM MgCl_2_ (Qiagen, Hilden, Germany), 10 µM of forward and reverse primers each, and finally 5 µL of nematode-extracted DNA. We used the following PCR conditions: 5 min 95 °C for initial denaturation, 40 cycles of 40 s 95 °C, 40 s 50 °C, 1 min 72 °C, and 10 min 72 °C for final extension. Purification of PCR products was performed using the commercial NucleoSpin® kit (Macherey–Nagel, Düren, Germany). The purified DNA was then diluted to final concentration of 10 ng/µl and sequenced by an external service provider (Microsynth AG, Switzerland). Out of 13 samples, we obtained five partial mtDNA *cox1* sequences which were further analyzed by the nucleotide Basic Local Alignment Search Tool (BLAST) search against other nematode sequences found in GenBank database.

### Statistical analysis

In this study, prevalence, mean intensity (mI), and mean abundance (mA) of anisakid larvae were determined according to Bush et al. (1997). In prevalence, CI was calculated by using Clopper-Pearson interval, and for mI and mA, CI was calculated by using bias-corrected and accelerated bootstrap (BCa bootstrap). Significance levels of differences between prevalence were calculated with Fisher’s exact test. The association between total mA of larvae with fish size was evaluated for each fish species with the Spearman’s coefficient. The significance level was set at *p* < 0.05. Statistical analysis was performed by Quantitative Parasitology QPweb (Reiczigel et al. 2019) and GraphPad® Prism software 2.0.

## Results

### Prevalence and levels of infection

**Fig. 1:**
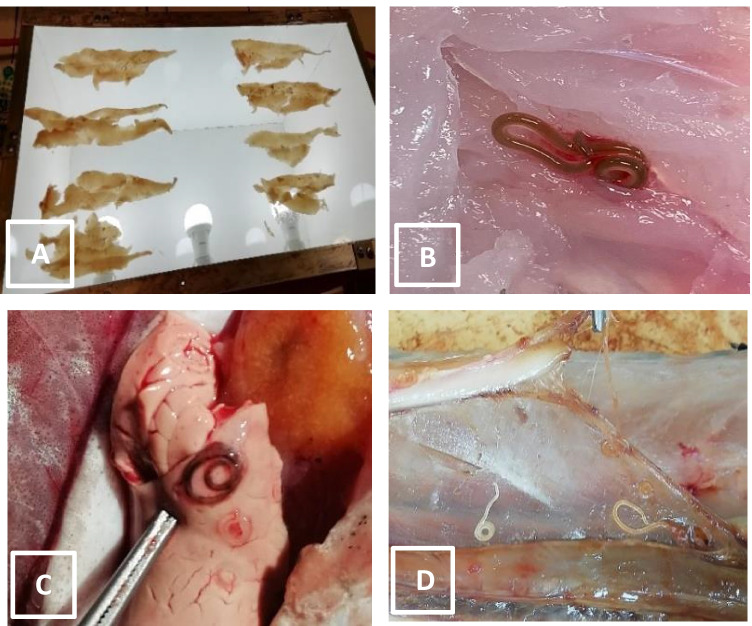
Parasitological analysis. **A** Image of parasitological analysis of muscle slices of sea breams (*Brama australis*) by trans-illumination technique. **B** *Pseudoterranova* third-stage larvae (L3) in muscle of Chilean hake (*Merluccius gayi*).**C** *Pseudoterranova* third-stage larvae (L3) in the liver of snoek (*Thyrsites atun*). **D** *Anisakis* larvae (L3) in peritoneum of an infected snoek (*Thyrsites atun*) covering abdominal organs

**Fig. 2:**
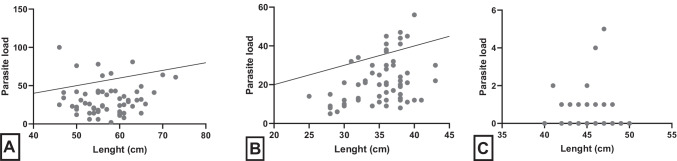
Determination of possible correlation between *Anisakis* spp. abundance and fish size on the analyzed fish species. **A** *Thyrsites atun*, *r* = 0.04; **B** *Merluccius gayi*, *r* = 0.41; **C** *Brama australis*, *r* = 0.01

In the present study, prevalence results showed a 100% prevalence of anisakid L3 in Chilean hakes (*M. gayi*) and snoeks (*T. atun*), followed by sea breams (*B. australis*) with a prevalence of 35% (Table 1). In addition, mI and mA resulted in high values for investigated Chilean hakes and snoeks highlighting the fact that all fish examined were infected with anisakid larvae (Table 1, Fig. 1). For sea bream, mI and mA were much lower indicating a very low parasite burden within infected individuals (mI), and with reference to all fish here examined (mA) compared with the rest of fish species analyzed (Table 1). Meanwhile, scatter plot demonstrated a significant positive correlation (*r* = 0.41; *p* < 0.01) via linear regression between anisakid larval abundance and fish size in Chilean hakes (see Fig. 2). In contrast, negative correlation was observed for snoeks (*r* = 0.04) and sea breams (*r* = 0.01; see Fig. 2).

**Table 1:** Morphometric data and epidemiological parameters of infection in fish examined. *N*_*Tot*_, number of larvae of *Anisakis* spp. collected; *mI*, mean intensity of infection; *mA*, mean abundance. Values within parentheses represent range; ^#^values within parentheses represent 95% confidence interval. In prevalence, CI was calculated by using Clopper-Pearson interval and in mI and mA, CI was calculated by using bias-corrected and accelerated bootstrap (BCa bootstrap)

Host	N	Mean length (cm)	Mean weight (g)	Prevalence (%) and CI^#^	*N*_Tot_	mI^#^	mA^#^
*Merluccius gayi*	60	35.12 (25–43)	325.20 (183–580)	100 (0.94–1.0)	1301	21.7 (18.8–24.8)	21.7 (19–25.1)
*Thyrsites atun*	60	57.13 (46–73)	1397 (940–2160)	100 (0.94–1.0)	1918	32 (27.5–37.8)	32 (27.3–37.7)
*Brama australis*	60	45.15 (40–50)	905 (590–1460)	35 (0.23–0.48)	30	1.43 (1.1–2.06)	0.5 (0.3–0.78)

Furthermore, comparison of anisakid-derived occurrence was determined according to location either in body cavity or in muscle observing significant differences in occurrence between body cavity and muscles in all examined fish (*p* < 0.0001; Table 2). Accordingly, in all internal organs analyzed, the highest occurrence of anisakid larvae was found in peritoneum for snoeks and sea bream (Table 3) followed by intestine in snoeks. While in Chilean hakes, highest occurrence was found in liver. The stomach was also analyzed showing higher occurrence on Chilean hakes, followed by snoeks and finally sea bream (Table 3). Interestingly, the gonads were infected only in Chilean hakes with high occurrence over 80%.

**Table 2:** Analysis of prevalence and mean intensity on muscle and body cavity of the examined fish. *mI*, mean intensity of infection; ^#^values within parentheses represent 95% confidence interval. In prevalence, CI was calculated by using Clopper-Pearson interval; in mI and mA, CI was calculated by using bias-corrected and accelerated bootstrap (BCa bootstrap). ^§^Significance level of differences between prevalence (Fisher’s exact test); *P < 0.05; **P < 0.001; ***P < 0.0001; *ns*, not significant

Host	Prevalence (%)^#^		mI^#^			*P*^§^
	Muscle	Body cavity		Muscle	Body cavity	
***Merluccius gayi***	18.6 (0.097–0.309)	100 (0.94–1.0)		1.73 (1.18–2.27)	21.4 (18.6–24.9)	***
***Thyrsites atun***	15 (0.07–0.26)	100 (0.94–1.0)		2 (1.44–2.56)	31.7 (27.2–37.7)	***
***Brama australis***	1.7 (0–0.089)	33 (0.21–0.46)		1	1.45 (1.1–2.1)	***

**Table 3:** Values of prevalence (%) and mean intensity (mI) of infection among internal organs of *Thyrsites atun* (snoeks), *Brama australis* (sea breams), and *Merluccius gayi* (Chilean hakes)

Location	*Thyrsites atun*		*Brama australis*		*Merluccius gayi*	
	Prevalence (%)	mI	Prevalence (%)	mI	Prevalence (%)	mI
Muscle	15	2	1.67	1	18.33	1.73
Liver	21.6	2.2	3.33	1	96.67	7.79
Stomach	45	4	8.33	1.4	48.33	2.97
Intestine	98.3	18.2	1.67	1	8.33	5.4
Gonads	0	0	0	0	86.66	7.33
Peritoneal cavity	100	11.45	23.33	1.36	83.33	6.72

**Table 4:** mtDNA *cox1* sequence analysis and species identification of larvae collected from the examined fish species.

Sample #	GenBank accession number	Host	cox1 sequence length	Query coverage	Pairwise identity	*E* value	GenBank ID	Species identified
5885	MW546052	Chilean hake	326 bp	100%	99.40%	8.06E-164	MT941432, KF545951	*Pseudoterranova cattani*
5886	MW546051	Chilean hake	311 bp	100%	98.10%	6.04E-150	MK228728, MK228723, MK228719, MK228710	*Anisakis pegreffii*
5887	MW546050	Snoek	340 bp	100%	100%	4.98E-176	NC_031644, KU558721	*Pseudoterranova cattani*
5889	MW546049	Snoek	339 bp	100%	99.90%	2.31E-174	MK228717, MK228716, LC222461	*Anisakis pegreffii*
5891	MW546048	Sea bream	321 bp	100%	98.90%	6.17E-160	MK228728, MK228723, MK228712, MK228710, (AF096226)	*Anisakis* sp.

### Molecular analyses of anisakid larvae

The sequences generated in this study are available in GenBank under accession numbers MW546048, MW546049, MW546050, MW546051, and MW546052 and the results from the BLAST search can be found in Table 4. Comparison to sequences previously deposited in GenBank indicated that larvae collected from two Chilean hakes had the highest sequence similarity with *P. cattani* (pairwise identity of 99.4%) and with *A. pegreffii* (pairwise identity of 98.1%) Table 4.

For the two snoek-derived larvae, *P. cattani* (pairwise identity of 100.0%) and *A. pegreffii* (pairwise identity of 99.9%) were identified. The larva found in sea bream had the highest pairwise identities to several GenBank entries assigned to *A. pegreffii* (pairwise identity of 98.9%) and with a single GenBank entry, deposited to *A. simplex* (pairwise identity of 98.9%) (AF096226). Nonetheless, since pairwise comparison is not conclusive, we have assigned them as *Anisakis* sp.

## Discussion

In the present study, a total of 180 freshly obtained fish specimens belonging to three highly commercialized species, namely Chilean hakes, snoeks, and sea breams, were examined for the presence of infective anisakid L3. Parasite infection parameters including prevalence, mI, and mA were estimated. Overall, all examined Chilean hake and snoek specimens were parasitized with infective anisakid L3, representing a 100% prevalence, whereas sea breams were the least infected host species showing 35% prevalence and a very low mI and mA values (1.43 and 0.5, respectively). Interestingly, mI and mA values resulted to be higher in snoeks (32.3) compared to Chilean hakes (21.7), a common fish species showing high anisakid prevalence worldwide (Valero et al. 2006; Casti et al. 2017). In this regard, composition of endoparasite fauna in marine organisms, including fish, is mainly influenced by their feeding habits (predator–prey relationships; Chavez et al. 2012). In Chile, fish preys of snoeks and Chilean hakes have also been reported to be infected with anisakid larvae indicating that these two piscivorous species could clearly act as paratenic hosts in epizootiology of anisakiasis (Valdivia et al. 2007; George-Nascimento and Moscoso 2013; Silva et al. 2020) being also trophically transmitted among diverse marine species (Valdivia et al. 2007). More importantly, Chilean hakes are one of the main pray fish of South American sea lions (*Otaria flavescens*) in this region, thereby probably representing a pivotal role transmission routes of anisakiasis (George-Nascimento and Llanos 1995; González-Saldía et al. 2015) and contributing to the perpetuation of life cycle. However, the role of snoeks and sea breams as intermediate and/or paratenic hosts as well as marine invertebrates in the biology of these parasites in this Chilean region has not yet been fully clarified.

Commonly, anisakid larvae parasitize viscera and/or musculature of intermediate host fish (Mattiucci and Nascetti 2008) but migration into flesh may also occur post mortem (Cipriani et al. 2016). In Chile, this last issue represents a serious public health concern since evisceration of fish is not always adequately controlled neither during artisanal fishing on vessels nor in local markets. As such, anisakid prevalence in muscle resulted in rather high levels with 18.6%, 15%, and 1.7% in Chilean hakes, snoeks, and sea breams, respectively. Prevalence of anisakid larvae in muscles of Chilean hakes has also been reported high reaching values of 24.3% (Silva et al. 2020) and 32% (Madrid et al. 2016) in Central-Southern regions and 29.2% in Southern regions of the country (Torres et al. 2000a, b) being relatively similar to our data. Conversely, prevalence of anisakid larvae in muscle tissue of snoeks and sea breams has scarcely been studied. Based on our data, only sea breams, which show almost negligible prevalence and mI (1.7 and 1, respectively) in muscles, may be considered partially “safe fish” species, therefore representing a lower risk for human anisakiasis but this needs further clarification due to our low number of examined fish. Irrespectively, food safety precautions and more accurate fish sanitary inspections should be taken into account for snoeks and Chilean hakes. More importantly, snoeks are very popular and frequently consumed as raw meat in this country and other South American countries as well. Therefore, control measures for avoidance of human anisakiasis are strongly recommended to national public health authorities.

In the present study, snoeks showed clearly much larger sizes and weight averages when compared to much smaller Chilean hakes and sea breams thereby augmenting the probability of larval accumulation as apex predators. Nevertheless, significant correlations on fish sizes and parasite load were only observed in Chilean hakes (*r* = 0.41; *p* < 0.01) being coincident to the same analysis on close related species, namely Atlantic hakes (*Merluccius merluccius*), from studies performed in the Northern hemisphere (Casti et al. 2017).

Regarding anisakid species spectrum, molecular analyses confirmed the presence of *P. cattani* and *A. pegreffii* in Chilean hakes, and *P. cattani* and *A. pegreffii* in snoeks. The larva found in sea bream was assigned as *Anisakis* sp. since pairwise comparison in this case was not conclusive. However, we propose a second multilocus analysis on those larvae for future research. In addition, our molecular analyses are limited by the use of a single, short fragment of the cox1 gene for molecular identification of the anisakid larvae. Analysis of additional polymorphic markers, such as the ribosomal internal transcribed spacer region, would improve identification of *A. simplex* s.s. and *A. pegreffii*. Adults of *P. decipiens* (sensu lato) are worldwide distributed as gastrointestinal nematodes of phocid and otariid seals comprising six biological species, genetically detected for the first time by allozymes (Paggi et al. 1991). *P. cattani* has also been found in fish from Argentinian waters in areas where colonies of South American sea lions (*O. flavescens*) are also present, in line to the present work (George-Nascimento and Urrutia 2000; Túnez et al. 2007; Feijoo et al. 2011).

Here, we demonstrate a new suitable intermediate host fish species hosting *P. cattani*, namely snoeks (*T. atun*). Concerning other zoonotic anisakid species, *A. pegreffii* was also detected in this study, where main definitive hosts are constituted by the family Delphinidae, Monodontidae, and Phocoenidae and, in less frequence, in Neobalenidae whales (Mattiucci and Nascetti 2006; Mattiucci et al. 2018). In all three fish species here studied, *A. pegreffii* was indeed found and this result is highly relevant since no records existed before on *A. pegreffii* infections in central coastal fish from Chile destined to human consumption. *A. pegreffii*, previously indicated as *A. simplex* A (Nascetti et al. 1986), is mainly distributed in the Mediterranean Sea and in the Southern hemisphere (Abollo et al. 2003) including South African coast, South Pacific Ocean (New Zealand), and Falkland Islands (Mattiucci and Nascetti 2007) and in the Austral Region between 30° S and 60° S, both in the larval and adult stages (Mattiucci et al. 2018; Bello et al. 2020). It is also widespread in the Pacific Boreal region including Japan Sea and China Sea (Gomez-Mateos et al. 2020; Gomes et al. 2021). Moreover, the genetic homogeneity between Mediterranean populations and those from Austral region seems to be maintained by the high levels of gene flow observed in this species allowing the hypothesis of its wide occurrence also in other areas of the Southern hemisphere (Mattiucci and Nascetti 2006; Mattiucci et al. 2018).

Among the *A. simplex* complex, *A. simplex* (s.s) have been found in subarctic and temperate waters of the Northern hemisphere. It has also been recorded in Western and Eastern Atlantic and Pacific Oceans likewise occurring with the biological species *A. berlandi* (former *A. simplex* C; Mattiucci et al. 2018) which exhibits a discontinuous distribution, being reported in the Southern Region, as in the Chilean Pacific Ocean, the South Shetland Islands, New Zealand waters, and the South African Atlantic coast (Mattiucci and Nascetti 2008; Mattiucci et al. 2018; Bello et al. 2021). From these geographical areas, *A. berlandi* has been identified at adult stage in six cetacean species, while its type I larvae have been, so far, identified in fish species from Southern waters from off New Zealand (Mattiucci et al. 2014, 2019; Bello et al. 2020), as well as from Southern Chilean (Mattiucci et al. 2018) and Argentine (Irigoitia et al. 2018) coasts.

Overall, by performing molecular analysis, we here demonstrated the presence of *P. cattani* jointly with *A. pegreffii* in three commonly consumed fish of Chile and other South American countries (Cabrera and Trillo-Altamirano 2004; Castellanos et al. 2018). These data are relevant for epidemiological and public health aspects considering the existing migration throughout Pacific Chilean coasts of several cetaceans and fish to Southern waters and the existing resident colonies of various sea lion species (i.e., South American sea lion (*O. flavescens*), South American fur seal (*Arctocephalus australis*), Juan Fernández fur seal (*Arctophoca philippii*)), underlying the complexity of anisakid life cycle for this particular geographic region. Prevalence of zoonotic anisakid larvae in meat of snoeks and Chilean hakes represents a public health hazard to Chilean population due to its culinary preferences in eating raw preparations. The current data should be considered a baseline study for future monitoring studies on anthropozoonotic anisakids circulating in wild fish, marine mammals, and their possible impact not only on public health issues but also on protected marine wildlife of Chile.
